# Use of tissue doppler imaging for the early detection of myocardial dysfunction in patients with the indeterminate form of Chagas disease

**DOI:** 10.1590/0037-8682-0457-2019

**Published:** 2020-02-21

**Authors:** Tomás Francisco Cianciulli, María Cristina Saccheri, Alonso Papantoniou, Ricardo José Méndez, Juan Alberto Gagliardi, Nilda Graciela Prado, Adelina Rosa Riarte, Luis Alberto Morita, Javier Eduardo Clérici, Jorge Alberto Lax

**Affiliations:** 1 Division of Cardiology, Hospital del Gobierno de la Ciudad de Buenos Aires “Dr. Cosme Argerich”, Argentina.; 2 Instituto Nacional de Parasitología “Dr. Mario Fatala Chaben” , Buenos Aires, Argentina.; 3 Researchers of the Ministry of Health of the Government of the City of Buenos Aires, Argentina.

**Keywords:** Indeterminate-form Chagas disease, Tissue Doppler imaging, Early detection of myocardial damage, Prognosis and treatment

## Abstract

**INTRODUCTION:**

Chagas disease is one of the most common diseases in Latin America and heart involvement is the main cause of death. This study aimed to determine differences in tissue Doppler imaging (TDI) parameters in the assessment left and right ventricular function in patients with the indeterminate form of Chagas disease compared to those in healthy controls.

**METHODS:**

We compared 194 patients with the indeterminate form of Chagas disease to 72 age-matched healthy individuals. We considered p-values <0.05 to be statistically significant.

**RESULTS:**

TDI analysis of the right ventricular (RV) showed lengthened isovolumic relaxation time (IRT) and higher RV index of myocardial performance (RIMP) and left ventricle (LV) index of myocardial performance (LIMP) in the Chagas group than in the control group, indicating RV and LV systolic and diastolic myocardial damage. TDI analysis of the myocardial velocities of the interventricular septum and the lateral wall of the LV also showed a systolic and diastolic myocardial damage.

**CONCLUSIONS:**

The study results demonstrated early LV systolic and diastolic myocardial damage in the RV and LV in patients with the indeterminate form of Chagas disease by TDI. These early findings of RV and LV dysfunction may help identify patients who will progress to heart failure during the disease course. TDI should be included in initial patient evaluations because it allows adequate follow-up and treatment.

## INTRODUCTION

Chagas disease was discovered more than 100 years ago and remains one of the most significant public health challenges in Latin America^1^ ^,^ ^2^. The World Health Organization estimates that 8 to 10 million people are infected worldwide and 100 million people are at risk, mostly in Latin America where the disease is endemic^2^ ^,^ ^3^ ^,^ ^4^ ^,^ ^5^. Moreover, Chagas disease causes approximately 50,000 deaths every year in Latin America, 60% of which are sudden^4^ ^,^ ^5^. The treatment of the complications of Chagas disease such as heart failure and the implantation of anti-arrhythmic devices makes it one of the costliest among so-called “neglected tropical diseases.” As much as 13% of the populations of the 21 endemic countries remain at risk of Chagas disease. The estimated national infection is highest in Bolivia (6.1%), followed by Argentina (3.6%) and Paraguay (2.1%), while the largest numbers of people living with Chagas disease, 42% of all cases, live in Argentina (1.5 million people) and Brazil (nearly 1.2 million people). Almost 1.2 million people in these countries likely have Chagasic cardiomyopathy.

In past decades, Chagas disease has also been detected in non-endemic countries, a phenomenon linked to population mobility and migratory movements that has led to the globalization of the disease^1^ ^,^ ^6^ ^,^ ^7^. As a consequence of global migration^3^ ^,^ ^8^, more than 300,000 individuals in the United States, 100,000 in Europe, 5,500 in Canada, 3,000 in Japan, and 1,500 in Australia are currently living with *Trypanosoma cruzi* infection. However, the disease is significantly underdiagnosed due to factors such as lack of clinician experience in detecting the disease, limited screening programs, and delayed diagnosis of the chronic phase of the disease because it remains largely asymptomatic for years. Many patients with Chagas disease are unaware of their infection status and can transmit the parasite through blood or organ donation^2^ ^,^ ^6^ ^,^ ^9^.

Chagas disease includes acute and chronic phases^8^. The chronic phase is divided into an indeterminate form, defined as the absence of clinical, radiological, and electrocardiography (ECG) abnormalities in a patient with serological positivity, as well as cardiac, digestive, and cardio-digestive forms with cardiac or digestive abnormalities. 

Tissue Doppler imaging (TDI) is a very sensitive echocardiographic tool used to detect systolic and diastolic dysfunction in both ventricles for several heart diseases and assess left ventricular (LV) filling pressure^10^ ^,^ ^11^ ^,^ ^12^. The major predictor of mortality and morbidity in patients with Chagas disease is LV dysfunction, and the prognosis is very poor once heart failure occurs^3^ ^,^ ^11^ ^-^ ^17^. Consequently, early detection of myocardial damage^18^ is essential for the appropriate treatment of patients with Chagas disease, thus improving their quality of life and life expectancy. The use of TDI has become widespread in recent years and it shows promise as a tool for the early detection of systolic and diastolic abnormalities in both ventricles. This study identified differences in TDI-derived parameters to assess LV and right ventricular (RV) function in patients with the indeterminate form of Chagas disease compared to those in healthy controls.

## METHODS

### Ethics statement

This study protocol was approved by the Institutional Review Boards and the Ethics Committees of the Hospital of the Government of the City of Buenos Aires “Dr. Cosme Argerich” and the National Institute of Parasitology, Buenos Aires, Argentina “Dr. Mario Fatala Chaben.”

Blood samples were collected at the National Institute of Parasitology “Dr. Mario Fatala Chaben.”

Written informed consent was obtained from all adult individuals and the samples were decoded and de-identified before their use for research purposes. All analyzed patient data were anonymized. All procedures were performed in accordance with the ethical standards of the responsible committee on human experimentation (institutional and national) and with the Helsinki Declaration of 1964 and its later revisions. Informed consent was obtained from all patients prior to study participation.

### Population

This observational, transversal study obtained data from the records of 688 patients with Chagas disease who underwent echocardiographic study including two-dimensional echocardiography, Doppler transmitral flow velocity assessment, and TDI at the Hospital of the Government of the City of Buenos Aires “Dr. Cosme Argerich” between March 2011 and January 2017. After excluding 494 patients with the chronic stage of the disease with heart disease or digestive abnormalities, we included 194 patients with the indeterminate form of Chagas disease and compared them to 72 age-matched healthy control individuals.

Patients with the indeterminate form of Chagas disease were defined as those with normal findings on clinical examination, ECG, chest plain radiography, and two-dimensional echocardiographic study. 

At recruitment, the patients underwent a standardized physical examination, including 12-lead ECG, chest plain radiography, and measurement of anti-*T. cruzi* antibody levels. Chagas disease was diagnosed as positivity for at least two of the following: enzyme-linked immunosorbent assay (positive >1:200), indirect hemagglutination (positive >1:32), and indirect immunofluorescence assays (positive >1:32). 

### Exclusion criteria

To minimize possible confounders or errors, the following exclusion criteria were applied: hypertension (systolic blood pressure ≥140 mm Hg or diastolic blood pressure ≥90 mm Hg), diabetes mellitus (glycated hemoglobin ≥6.5%, fasting glucose ≥126 mg/dL, or 2-h glucose ≥200 mg/dL), anemia (hemoglobin concentration <120 g/L in women and <130 g/L in men), asthma (widespread airway obstruction reversible over short periods, either spontaneously or following treatment), chronic obstructive pulmonary disease (airflow limitation that is not fully reversible and usually progressive with some significant extrapulmonary effects), alcoholism (more than four drinks per day in men or more than three drinks per day in women), obesity (body mass index ≥30 kg/m^2^), current smoking (smoking part or all of a cigarette during the 30 days preceding the survey and reported lifetime cigarette use ≥100 cigarettes), pregnancy, a previous history of tuberculosis or thyroid dysfunction, renal failure, known coronary artery disease, congenital heart disease, cardiomyopathies, moderate or severe valvular heart disease, pericardial disease, and atrial fibrillation.

### Control group

The control group included 72 healthy individuals who were negative for *T. cruzi* serology and had normal findings on clinical examination, ECG, chest plain radiography, and two-dimensional echocardiography.

### Two-dimensional echocardiography

Doppler-echocardiography was performed using a Vivid 7 instrument (GE Healthcare, Wauwatosa, WI, USA) with a 1.5- to 4-MHz transducer. Apical views (two-chamber, four-chamber, and long-axis) and parasternal views (short- and long-axis) were used.

M-mode and two-dimensional echocardiographic images were acquired from the short-axis view at the papillary muscle level according to American Society of Echocardiography and European Association of Echocardiography guidelines^19^. The LV end-diastolic diameters (LVEDds), LV end-systolic diameters (LVESds), and septal and posterior wall thickness parameters were also obtained. The anteroposterior diameter of the left atrium (LA) was obtained using the left parasternal long-axis view. The fractional shortening of the LV was calculated using the formula LVFS = [(LVEDd-LVESd)/LVEDd] × 100. 

Two-dimensional LV volumes and the LV ejection fraction (LVEF) were calculated using the modified Simpson rule (biplane method), with images acquired from the apical four- and two-chamber views. Three consecutive cardiac cycles in each view were digitally stored for subsequent offline analysis. All reported results were the average of three cardiac cycles. The LVEF was calculated using the following formula LVEF = [(EDV-ESV)/EDV] × 100, where EDV is the end-diastolic volume and ESV is the end-systolic volume. An abnormal LVEF was defined as <55%.

The LV mass index was estimated using Devereux’s formula. Hypertrophy was defined as >115 g/m^2^ for men and >95 g/m^2^ for women.

Left atrial volume was measured using the biplane method of disks from the apical four- and two-chamber views at the ventricular end-systole (maximum LA size). LA volume >34 mL/m^2^ was considered dilated.

### Transmitral flow velocity

The transmitral flow velocity and LA dimension were used to evaluate global diastolic function. The peak early diastolic velocity (E wave), peak late diastolic velocity (A wave), E/A ratio, and early filling deceleration time were obtained from the four-chamber apical view by placing the sample volume at the tip of the mitral valve leaflets. 

### TDI

TDI was performed using a 1.5- to 4-MHz transducer. The longitudinal right and left annulus motions were recorded using color-guided pulse-wave tissue Doppler from the apical four-chamber view. The sample volume was placed at the septal borders of the mitral annulus and the basal-lateral walls of both ventricles through a four-chamber apical view.

The LV systolic and diastolic functions were assessed and measured at peak systolic myocardial velocity (s´), early diastolic myocardial velocity (e´), late diastolic myocardial velocity (a´), isovolumic relaxation time (IRT), isovolumic contraction time (ICT), and isovolumic contraction time peak velocity (ICT peak velocity).

Pulsed-wave TDI imaging was performed with the sample volume at the septal mitral annulus to obtain the average peak longitudinal early diastolic annular (e′) velocity, which was used to calculate the E/e′ ratio by dividing the peak early wave of transmitral flow by the early diastolic myocardial velocity determined from TDI.

**FIGURE 1:**
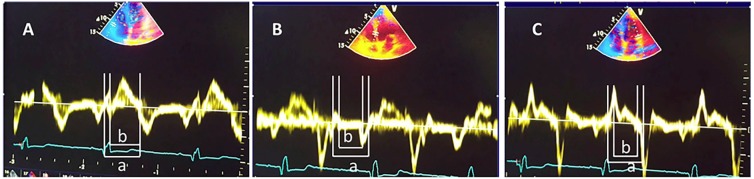
Tissue Doppler myocardial performance index measurement. Samples taken from the lateral annulus of the tricuspid valve **(A)**, interventricular septum **(B),** and lateral wall of the left ventricle **(C).** Note the lack of isovolumetric relaxation in the right ventricle. a = onset of isovolumic contraction time to the end of isovolumic relaxation time. b = ejection time. Right ventricle index of myocardial performance (RIMP) and left ventricle index of myocardial performance (LIMP) were calculated using the formula (a-b)/b.

RV and LV systolic functions were also assessed using the TDI RV index of myocardial performance (RIMP) and the LV index of myocardial performance (LIMP). TDI RIMP and LIMP were calculated using the formula (a-b)/b, where *a* was the onset of isovolumic contraction time to the end of IRT and *b* was the ejection time (Figure 1).

### Statistical analysis

Qualitative variables were described using absolute and relative frequencies and were compared using chi-squared tests. Kolmogorov-Smirnov tests were used to verify the normality distribution of the continuous variables. Normally distributed data were presented as means ± standard deviation, while non-Gaussian distribution data were presented as medians (interquartile intervals). Unpaired t-tests were used to compare intergroup quantitative variables with normal distributions, while Wilcoxon rank-sum tests were used to compare variables with non-normal distributions. Statistix 7.0 and Epi-Info 2008 version 3.5.1 were used to perform the analyses. P values <0.05 were considered significant

### Intra-observer and inter-observer variability

Offline TDI measurements were performed by a single observer (TFC) blinded to the clinical features of the 11 patients in the study cohort. The 11 studies were analyzed by another observer (JAL) to determine the inter-observer variability. The same observer (JAL) reanalyzed the same 11 studies one hour later to assess intra-observer variability. Intra- and inter-observer agreements were measured by intraclass correlation coefficients.

## RESULTS

We compared 194 patients with the indeterminate form of Chagas disease (Chagas group) to 72 healthy individuals (control group). 

### Echocardiographic flow findings

Table 1 shows clinical and echocardiographic findings (M mode and 2-D echocardiogram). 

**TABLE 1:** Clinical and echocardiographic findings (M mode and two-dimensional echocardiogram).

**Variable**	Control group	Chagas group	p value
	(n=72)	(n=194)	
Age (y)	40.9 ± 11.9	41.9 ± 8.4	0.34
Female, n (%)	22 (30.6)	100 (51)	0.002
Body mass index (kg/m^2^)	25.3 ± 3.03	26.9 ± 4.52	0.06
Heart rate (BPM)	70 ± 9	71 ±10	0.46
Systolic blood pressure (mmHg)	121 ± 7	119 ± 6	0.06
Diastolic blood pressure (mmHg)	78 ± 8	77 ± 7	0.42
LVEDd (mm)	49.3 ± 4.4	48.1 ± 4.5	0.052
LVESd (mm)	27.8 ± 4.6	28.3 ± 4.0	0.35
LVFS (%)	43.7 ± 6.6	45,9 ± 6.4	0.06
IVS (mm)	9.7 ± 1.43	9.7 ± 1.64	0.90
LVPW (mm)	7.54 ± 1.02	8.11 ± 1.08	0.06
LV mass index (g/m^2^)	92.51 ± 16.8	95.24 ± 19.9	0.30
LA (mm)	35.1 ± 3.7	35.4 ± 4.3	0.51
LA (mL/m^2^)	30.2 ± 3.80	30.1 ± 3.9	0.85
Aortic root (mm)	30.1 ± 3.4	30.8 ± 3.7	0.21
LVEDV (mL/m^2^)	52,1 ± 3.2	53,3 ± 3,1	0.06
LVESV (mL/m^2^)	20,3 ± 3,3	21,2 ± 3,2	0.06
LVEF (%)	60,3 ± 4,1	62,3 ± 2,1	0.06

Values are expressed as absolute numbers (%) and means ± SD. **LVEDd:** left ventricular end diastolic dimension, **LVESd:** left ventricular end systolic dimension, **LVFD**: left ventricular fractional shortening, **IVS**: interventricular septum, **LVPW:** left ventricular posterior wall, **LA:** left atrial dimension, **LV:** left ventricle; **LVEDV:** left ventricular end diastolic volume; **LVESV:** left ventricular end systolic volume; **LVFS:** left ventricular fractional shortening; **LVEF:** left ventricular ejection fraction; **BPM:** beat per minute.

Patients with the indeterminate form of Chagas disease were predominantly women and had a higher body mass index, LVEDV and LVFS. Systolic blood pressure and LVEDd were lower in patients with Chagas disease. 

### Transmitral and transtricuspid Doppler flow findings

Table 2 shows statistically significant differences in transmitral A peak velocity and mitral E/A ratio between the two groups, indicating an early disorder of LV diastolic function in patients with the indeterminate form of Chagas disease.

**TABLE 2:** Transmitral and transtricuspid Doppler flow parameters.

Variable	Control group	Chagas group	p value
	(n=72)	(n=194)	
Mitral E wave (cm/sec)	68 (61- 83)	69 (58-81)	0.49
Mitral A wave (cm/sec)	48 (42-58)	54.5 (47-64)	0.002
Mitral E/A ratio	1.44 (1.18-1.68)	1.22 (1.00-1.53)	0.001
Mitral DT (msec)	163.0 ± 52.3	173.4 ± 44.1	0.11
Tricuspid E wave (cm/sec)	50 ± 11	45 ± 13	0.09
Tricuspid A wave (cm/sec)	38 ± 8	34 ± 11	0.15
Tricuspid E/A ratio	1.36 ± 0.25	1.39 ± 0.41	0.70
Tricuspid DT (msec)	215.8 ± 59.5	185.7 ± 63.1	0.06

**E**: peak early diastolic velocity, **A**: peak late diastolic velocity, DT: early filling deceleration time.

Analysis of the variables measured in transtricuspid flow did not show significant differences between groups.

### Intra- and inter-observer agreement of transmitral and transtricuspid Doppler flow measurements

Intra-observer agreement measured using the intraclass correlation coefficient varied between 0.995 for the velocities and 0.882 for the time measurements. Inter-observer agreement varied between 0.993 and 0.832, respectively. 

### Tissue Doppler imaging findings

Table 3 shows the comparative analysis of the TDI parameters of the lateral wall of the RV and the lateral wall and interventricular septum (IVS) of the LV in both groups. 

**TABLE 3:** Tissue Doppler Imaging findings.

Variable	Control group	Chagas group	p value
	(n=72)	(n=194)	
***Lateral basal wall of the RV***			
s´ (cm/sec)	13.4 ± 3.52	13.8 ± 2.27	0.96
ICT (msec)	80.46 ± 21.4	74.96 ± 17.3	0.03
IRT (msec)	45.4 ± 24.6	63.6 ± 27.6	0.0004
e´ (cm/sec)	13.5 (11.0-16.0)	13.0 (11.0-15.0)	0.50
a´ (cm/sec)	13.0 (10.0-16.5)	13.0 (11.0-15.0)	0.59
e´/a´ ratio	0.94 (0.77-1.33)	1.0 (0.79-1.29)	0.90
RIMP (sec x cm^-1^)	0.29 ± 0.24	0.40 ± 0.20	0.0003
Lateral E/e´ ratio	5 (4- 6)	6 (4-7)	0.46
ICT Peak Velocity (cm/sec)	12.0 (9.5-14.0)	12.0 (10.0-14.0)	0.88
***Lateral basal wall of the LV***			
s´ (cm/sec)	10.2 ± 3.6	10.3 ± 2.7	0.014
ICT (msec)	86.1 ± 25.6	78.8 ± 18.9	0.024
IRT (msec)	68.9 ± 23.4	75.3 ± 18.6	0.026
e´ (cm/sec)	13.9 ± 4.76	13.9 ± 3.8	0.65
a´ (cm/sec)	9.2 ± 3.5	10.9 ± 2.9	0.0002
e´/a´ ratio	1.5 (1.1-2.0)	1.3 (1.0-1.6)	0.0029
Lateral LIMP (sec x cm^-1^)	0.42 ± 0.41	0.49 ± 0.43	0.19
Lateral E/e´ ratio	5 (4-6)	5 (4-6)	0.77
ICT peak velocity (cm/sec)	6.37 ± 2.5	8.11 ± 3.5	0.06
***Interventricular basal septum***			
s´ (cm/sec)	8.9 ± 2.7	8.7 ± 1.8	0.03
ICT (msec)	76.9 ± 22.2	76.6 ± 19.6	0.91
IRT (msec)	70.9 ± 27.9	86.3 ± 22.9	0.0001
e´ (cm/sec)	11.1 ± 3.8	10.5 ± 2.7	0.03
a´ (cm/sec)	8.9 ± 2.91	9.5 ± 2.0	0.12
e´/a´ ratio	1.22 (1.09-1.75)	1.05 (0.83-1.37)	0.01
Septal LIMP (sec x cm^-1^)	0.52 ± 0.44	0.78 ± 0.39	0.0001
Septal E/e´ ratio	6 (5-7)	6.7 (6-8)	0.079
ICT peak velocity (cm/sec)	6.25 ± 2.0	7.34 ± 3.3	0.17

Values are expressed as absolute numbers (%), means ± SD, or medians (interquartile ranges).

**IVS:** interventricular septum, **s´:** myocardial contraction, **a´:** myocardial late diastolic velocity, **e´:** myocardial early diastolic velocity, **ICT:** isovolumic contraction time, **IRT:** isovolumic relaxation time, **RIMP:** right ventricular index of myocardial performance, **LIMP:** left ventricular index of myocardial performance, **E:** peak early diastolic flow velocity, **RV:** right ventricle, **LV:** left ventricle.

**FIGURE 2:**
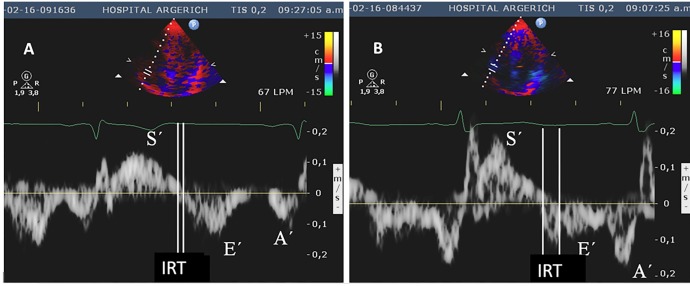
Doppler tissue imaging of the basal portion of the right ventricular free wall. **(A)** Normal control, **B)** Chagasic patient showing delayed isovolumic relaxation time. **S´:** Systolic myocardial wave; **E´:** early diastolic myocardial wave; **a´:** late diastolic myocardial wave; **IRT:** isovolumic relaxation time.

The IRT of the RVs was longer in the patients with Chagas disease (Figure 2). The RIMP, which evaluates both systolic and diastolic functions^20^, was higher in the Chagas group than in the control group These findings revealed mixed RV systolic and diastolic dysfunction in patients with Chagas disease. However, the s´ wave, which is also a parameter of systolic function, did not differ significantly between the groups. 

Evaluation of the lateral wall of the LV by TDI showed diastolic dysfunction of the LV but no systolic dysfunction in patients with Chagas disease. The IRT was prolonged, the ICT was shortened, and a higher myocardial late diastolic velocity was observed in the Chagas group than those in the control group. The e´/a´ ratio was lower in the Chagas group than in the control group, thereby revealing diastolic dysfunction. The s´ in the Chagas disease was normal compared to that in the control group. 

Similarly, analysis of the TDI parameters of the interventricular septum showed diastolic dysfunction in patients with Chagas disease, including prolonged IRT, lower myocardial early diastolic velocity (e´), and significantly lower e´/a´ ratio. Moreover, s´ was significantly lower in patients with Chagas disease than that in the control group, indicating LV systolic dysfunction. The high septal LIMP in the patients with Chagas disease revealed major LV systolic and end-diastolic dysfunctions.

The E/e´ ratio did not demonstrate increased LV end=diastolic pressure because there was no statistical difference between normal patients and patients with Chagas disease.

### Intra- and inter-observer agreements in TDI measurements

Intra-observer agreement measured using intraclass correlation coefficients varied between 0.985 for the velocities of both the ventricles and 0.889 for the time measurements. Inter-observer agreement varied between 0.987 and 0.892, respectively.

## DISCUSSION

Chagas disease is one of the most significant diseases in Latin America, and its cardiac manifestation is responsible for most deaths due to *T. cruzi* infection. After the acute infection stage, patients experience the indeterminate form of the disease, which is usually defined by the absence of clinical, ECG, chest plain radiographic, and two-dimensional echocardiographic abnormalities^1^ ^,^ ^3^ ^,^ ^4^ ^,^ ^16^. Nevertheless, many studies have demonstrated that patients with the indeterminate form of Chagas disease may have ventricular function abnormalities in Doppler transmitral flow velocity assessment or TDI^15^ ^,^ ^18^ ^-^ ^23^. In our previous investigation^18^, Doppler transmitral flow velocity helped to identify early abnormalities in LV diastolic function in patients with the indeterminate form of Chagas disease. 

To our knowledge, this is the first study to demonstrate a significantly elevated lateral LIMP in patients with the indeterminate form of Chagas disease. This finding suggests LV systolic dysfunction of the lateral wall. This dysfunction was documented initially and was associated with the isovolumic contraction time^22^ ^,^ ^24^. However, our findings indicate that it is also related to prolonged IRT and shortened ejection time, as demonstrated by the elevated LIMP of the lateral wall of the LV. The cardiac manifestations of Chagas disease begin as local myocarditis, after which the damaged tissue is replaced by fibrotic tissue. Therefore, even patients with the indeterminate form of Chagas disease may have early LV abnormalities that are not severe enough to cause a global systolic or diastolic dysfunction^8^ ^,^ ^13^ ^,^ ^22^ ^,^ ^23^. TDI measures myocardial changes during the cardiac cycle rather than the global dimension of the chambers or flow dynamics as do the other echocardiographic methods. TDI also is less influenced by changes in load and may, therefore, allow earlier detection of these focal abnormalities in different cardiac walls^4^ ^,^ ^21^ ^-^ ^24^.

The TDI findings in patients with the indeterminate form of Chagas disease were statistically more significant than the transmitral flow findings; therefore, TDI may be more sensitive for the detection of early myocardial damage.

Barros et al^21^ first reported that patients with the indeterminate form of Chagas disease evaluated using TDI had lengthened ICT in the septal wall indicative of LV dysfunction. Unlike Barros et al, our data did not show any significant differences in ICT; however, we observed increased IRT of the lateral wall of the LV and IVS, thus revealing early LV diastolic myocardial damage^21^ ^,^ ^22^. This finding can be explained by the fact that Chagas disease impairments first affect the relaxation phase by lengthening it. Ventricular stiffness is affected next, leading to a restrictive pattern; hence, some patients without abnormalities in compliance may show early relaxation abnormalities^21^ ^,^ ^25^. We also observed decreased e´ of the IVS and e´/a´ ratio of the LV and IVS. All these abnormalities demonstrated early LV diastolic myocardial damage to both walls^21^ ^,^ ^25^.

In patients with the indeterminate form of Chagas disease that present functional subclinical cardiac abnormalities, it is possible that the muscle fibers couple and develop the strength needed to eject the blood to the arterial bed. However, because of focal myocardial damage, this phase is not properly harmonized, resulting in delayed IRT^24^ ^,^ ^26^. 

This early detection of LV systolic and diastolic myocardial dysfunction is of interest since early findings of dysfunction in Chagas disease may help to identify patients who will progress to heart failure during the disease course.

As Barros et al^23^ noted, “the involvement of the RV in Chagas disease is early and frequent;” however, RV dysfunction is traditionally difficult to assess due to its anatomic features. However recent studies have demonstrated that TDI can correctly evaluate RV function^16^ ^,^ ^23^. Our analysis demonstrated lengthened RV IRT and elevated RIMP indicating both an early systolic and diastolic RV myocardial damage, even in patients with normal RV filling when pulsed by Doppler flow.

We did not use a reference method such as magnetic resonance imaging (MRI) to assess RV and LV functions because TDI is more commonly available than MRI in Argentina and other Latin American countries.

This study has some limitations. First, there were significant differences in the proportions of sexes between groups; thus, sex may be a confounding factor. Second, TDI parameters can be affected by the translation of the heart. Third, although this study detected RV dysfunction, it was impossible to know whether it preceded or was associated with LV dysfunction. This study also has limitations inherent to its cross-sectional design. Further studies are required to address these limitations. 

The results of this study revealed early LV systolic and diastolic myocardial damage in the RV and LV of patients with the indeterminate form of Chagas disease by TDI. This early detection is of particular interest since early findings of LV dysfunction in Chagas disease may help identify patients who will progress to heart failure during the disease course. Consequently, TDI is a useful tool to detect early abnormalities in these patients and should be included in the initial evaluation of Chagas disease to enable adequate follow-up and treatment.
